# A conserved viral amphipathic helix governs the replication site-specific membrane association

**DOI:** 10.1371/journal.ppat.1010752

**Published:** 2022-09-01

**Authors:** Preethi Sathanantham, Wenhao Zhao, Guijuan He, Austin Murray, Emma Fenech, Arturo Diaz, Maya Schuldiner, Xiaofeng Wang

**Affiliations:** 1 School of Plant and Environmental Sciences, Virginia Tech, Blacksburg, Virginia, United States of America; 2 Institute of Plant Protection, Jiangsu Academy of Agricultural Sciences, Key Lab of Food Quality and Safety of Jiangsu Province-State Key Laboratory Breeding Base, Nanjing, China; 3 Department of Molecular Genetics, Weizmann Institute of Science, Rehovot, Israel; 4 Department of Biology, La Sierra University, Riverside, California, United States of America; Purdue University, UNITED STATES

## Abstract

Positive-strand RNA viruses assemble their viral replication complexes (VRCs) on specific host organelle membranes, yet it is unclear how viral replication proteins recognize and what motifs or domains in viral replication proteins determine their destinations. We show here that an amphipathic helix, helix B in replication protein 1a of brome mosaic virus (BMV), is necessary for 1a’s localization to the nuclear endoplasmic reticulum (ER) membrane where BMV assembles its VRCs. Helix B is also sufficient to target soluble proteins to the nuclear ER membrane in yeast and plant cells. We further show that an equivalent helix in several plant- and human-infecting viruses of the *Alsuviricetes* class targets fluorescent proteins to the organelle membranes where they form their VRCs, including ER, vacuole, and Golgi membranes. Our work reveals a conserved helix that governs the localization of VRCs among a group of viruses and points to a possible target for developing broad-spectrum antiviral strategies.

## Author summary

Positive-strand RNA viruses [(+)RNA viruses] are the largest viral class that include numerous pathogens causing important diseases in humans, animals, and plants. During their infections, (+)RNA viruses assemble their viral replication complexes (VRCs), where they multiply themselves, at specific organelle membranes. An initial step to form VRCs is to target viral replication proteins to the designated organelle membranes. For brome mosaic virus (BMV), its replication protein 1a is responsible for the VRC formation at the nuclear endoplasmic reticulum (ER) membrane. We show that an amphipathic alpha-helix, helix B, in BMV 1a is necessary for the association of BMV 1a with the nuclear ER membrane and for BMV genome amplification. In addition, Helix B is sufficient to target several soluble proteins to the nuclear ER membrane in yeast and plant cells. BMV belongs to the *Alsuviricetes* class that includes viruses infecting humans, animals, and plants. We further show that the helix B across members of the *Alsuviricetes* class is sufficient to target fluorescence proteins to the designated organelle membranes. Our results reveal a conserved feature among a group of viruses in governing the associations with replication site-specific organelle membranes and point to a target to develop broad-spectrum antivirals.

## Introduction

Positive-strand RNA viruses [(+)RNA viruses] are the largest viral class, including many important human and animal pathogens as well as the great majority of plant viruses. Just a few examples include SARS-COV-2 that has infected more than 540 million and led to the death of >6.3 million people in the world [1]; foot and mouth disease virus, the most economically important animal virus; and cucumber mosaic virus (CMV), which infects more than 1,200 plant species. Despite very different host ranges, (+)RNA viruses are grouped into alphavirus-, flavivirus-, and picornavirus-like superfamilies, based on their genome organization and homology of RNA-dependent RNA polymerase [2]. Each superfamily includes viruses infecting humans, animals, and plants. The alphavirus-like superfamily is now recognized as the *Alsuviricetes* class, the flavivirus-like superfamily as the *Flasuviricetes* class, and viruses in the picornavirus-like superfamily are grouped into the classes of *Pisoniviricetes* and *Stelpaviricetes* (https://talk.ictvonline.org/taxonomy/).

A universal feature of (+)RNA viruses is that they assemble their viral replication complexes (VRCs) or replication organelles on specific cellular organelle membranes [3,4] and this is attributed to the membrane lipid compositions [5–7], among others. The viral replication proteins that commence membrane associations can either be a true integral membrane protein with transmembrane domain(s) (TMDs), such as protein A of Flock House virus (FHV) [8,9], or peripheral membrane-associated proteins such as 2C of poliovirus [10] and nSP1 (non-structural protein 1) of Semliki Forest virus (SFV) [11]. Targeting viral replication proteins to the designated organelle membranes is the initial step towards viral replication, and therefore, the knowledge of structural features responsible for protein targeting will not only gain a better understanding of viral replication mechanisms but also underlie our ability to develop new ways for viral control.

Brome mosaic virus (BMV) shares replication features with (+)RNA viruses from many other families and therefore has been useful to investigate fundamental aspects of viral replication and associated processes [12,13]. BMV is the type member of the family *Bromoviridae* and a representative member of the alphavirus-like superfamily that has been renamed as the *Alsuviricetes* class, which include several families of viruses infecting humans and animals, as well as a large number of plant virus families [13,14]. BMV has a tripartite genome where genomic RNA1 encodes replication protein 1a (1a herein), and RNA2 encodes 2a (2a^pol^ herein) that has a conserved RNA-dependent RNA polymerase domain. RNA3 encodes the movement protein 3a, and coat protein, which is expressed from RNA4, a subgenomic mRNA that is a replication product of RNA3 [14,15]. BMV assembles its spherular VRCs at the perinuclear ER (nER) membrane [12]. Independent of other viral components, 1a localizes to the nER membrane where it induces the invaginations of the outer nER member into the lumen to form spherules [12], which become VRCs when 2a^pol^ and viral genomic RNA templates are recruited by BMV 1a [12].

**Fig 1:**
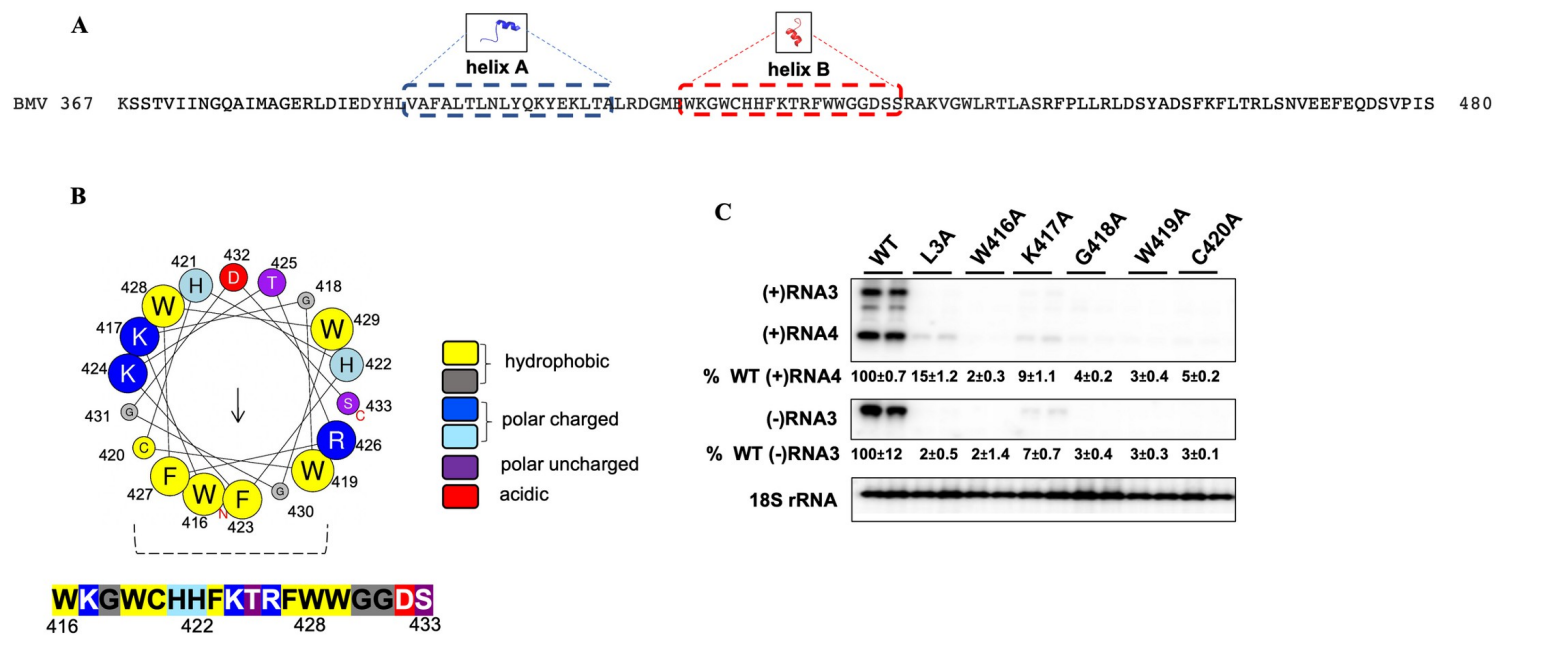
Mutations in BMV 1a helix B inhibit viral genome replication. **(A)** Helix A and helix B are highlighted in blue and red respectively and their amino acid sequences are shown. Note region E is indicated as a 114 amino acid-long sequence consisting of helix A and helix B. **(B)** Helical wheel projection generated using an 18 aa analysis window in Heliquest (https://heliquest.ipmc.cnrs.fr/). The predicted hydrophobic face is formed by W419, G430, F423, W416, F427, and C420, as indicated by the dotted lines. The size of each residue in the helical wheel represents the bulkiness of their sidechain and the arrow in the helical wheel corresponds to the hydrophobic moment. **(C)** BMV genomic replication was measured by using Northern blot for WT and various 1a mutants. Positive- and negative-strand viral RNAs were detected using BMV strand-specific probes. 18S rRNA served as a loading control. Band intensities were measured using Adobe photoshop and the standard deviations are indicated. All experiments were repeated at least three times in duplicates each time. Note: L3A has 3 Leu residues replaced by Ala in helix A and serves as a negative control.

BMV 1a has a capping domain at the N-terminus [16,17], a helicase-like domain at the C-terminus with a demonstrated NTPase activity [18], and a linker region enriched in prolines between two domains [14]. It has been previously revealed that a 114-amino acid region in 1a, region E [amino acids (aa) 367–480], is necessary for its nER membrane association [19]. Further studies have identified an amphipathic α-helix, helix A (aa 392–409, Fig 1A) within region E, that regulates 1a’s membrane association, as well as the size and frequency of VRCs, among others [20]. Although helix A was shown to be sufficient to target GFP to membrane fractions, it remained unclear whether helix A is sufficient to target proteins specifically to the nER membrane [20]. A second amphipathic α-helix, helix B (aa 416–433, Fig 1B), which is within region E and immediately downstream of helix A [21], has been shown to play a crucial role in membrane permeabilization to release the oxidizing potential of the ER lumen, and thus, maintain efficient BMV genome replication [22].

We report here that helix B is necessary for 1a’s nER membrane association and is sufficient to target several soluble proteins to the nER membrane, extending our understanding on the roles of helix B in targeting 1a to the designated organelle for the VRC formation. Furthermore, we show that the predicted helix B motifs from replication proteins of cowpea chlorotic mottle virus (CCMV), CMV, hepatitis E virus (HEV), and Rubella virus (RuV) direct soluble fluorescent protein(s) to specific organelles where these viruses replicate, revealing a functionally conserved motif across the *Alsuviricetes* class and pointing out a potential antiviral strategy to control a large group of viruses.

## Results

### Helix B of 1a is necessary for BMV RNA replication

For many membrane-associated proteins without TMDs, shallow insertion of amphipathic helices into membranes promotes membrane binding and remodeling [23,24]. BMV 1a does not have any TMD but is tightly associated with the membrane [19]. It has been previously reported that helix A was able to target GFP to membranes based on a membrane flotation assay [20], however, it was unclear whether helix A is sufficient for 1a’s association with the nER membrane. We tested whether helix B is involved in targeting 1a to the nER membrane or whether a concerted action by both helices is required.

We first tested whether helix B is necessary for viral replication by deleting the entire sequence of helix B to make the 1a-ΔB mutant. In cells expressing 2a^pol^, RNA3, and wild-type (WT) 1a, strong signals were detected for both (-)RNA3 and (+)RNA4, which can only be produced after a full round of replication (Fig 1C). In parallel with the previous report that deleting helix A blocked BMV replication [20], no (-)RNA3 or (+)RNA4 band was detected with the 1a-ΔB mutant, indicating that helix B is necessary for BMV replication (Table 1).

**Table 1:** Characteristics of 1a helix B mutants.

	1a or 1a mutants	(-)RNA3 accumulation (% of WT)	1a accumulation (% of WT)	1a-mC localization	nER-localized 1a-mC (% of total cells)
	WT	100	100±12	nER*	87 ± 3
**Group A**	1a-ΔB	0	104±13	C+phER^†^	0 (p<0.05)
	W416A	3±1 (p<0.05)	113±8	C+phER	7 ±2 (p<0.05)
	G418A	5±2 (p<0.05)	98±12	C+phER	0 (p<0.05)
	W419A	4±3 (p<0.05)	100±18	C+phER	2 ±1 (p<0.05)
	C420A	3±2 (p<0.05)	118±6	C+phER	0 (p<0.05)
	H422A	0 (p<0.05)	123±11	C+phER	0 (p<0.05)
	K424A	7±1 (p<0.05)	92±9	C+phER	0 (p<0.05)
	R426A	0 (p<0.05)	101±11	C+phER	12 ±2 (p<0.05)
	G431A	7±6 (p<0.05)	168±14	C+phER	7 ±2 (p<0.05)
**Group B**	K417A	9±2 (p<0.05)	98±12	nER	36 ±7 (p<0.05)
	H421A	4±1 (p<0.05)	96±7	nER	28 ±7 (p<0.05)
	W429A	4±1 (p<0.05)	94±8	nER	20 ±7 (p<0.05)
	G430A	4±1 (p<0.05)	89±12	nER	31 ±5 (p<0.05)
	D432A	9±7 (p<0.05)	164±15	nER	18 ±4 (p<0.05)
**Group C**	F423A	51±25 (p<0.05)	102±11	nER	57 ±9 (p = 0.004)
	T425A	28±1 (p<0.05)	112±9	nER	62 ±10 (p = 0.01)
	F427A	29±5 (p<0.05)	112±3	nER	20 ±7 (p<0.05)
	W428A	41±3 (p<0.05)	100±5	nER	27 ±8 (p = 0.0002)
	S433A	84±1 (p<0.05)	96±11	nER	70 ±6 (p = 0.004)

*nER represents the nuclear ER membrane for all mutants tested. However, the percentage of cells showing nER-localization across mutants was consistently low with respect to cells expressing WT 1a.

^†^cytosolic (C) and peripheral ER (phER) associated mutants.

Mutants are categorized into three groups, A, B, and C, based on their localization and the capacity to support viral genome replication. Note that only those mutants with >15% cells displaying nER localization are designated as nER. For the localization experiments, the percentage of cells represents the number of cells that colocalized with ER markers among at least 100 cells where visible signals of both mC and GFP were observed (Percentage = [(number of cells with colocalized GFP and mC signals at the inner ring)/(total number of cells with visible GFP and mC signals)*100]). The adjusted p-value calculated for localization of all mutants with respect to WT 1a was <0.05. Two independent experiments were performed to include all mutants for the replication and 1a accumulation analyses wherein each experiment consisted of duplicated samples. Note: the standard deviations in the 1a accumulation data were calculated by comparing the duplicates in a single western blot experiment.

We next substituted every amino acid in helix B with alanine and characterized each mutant on their ability to support BMV RNA replication. Most mutants failed to support genome replication, producing (-)RNA3 at levels less than 10% of that with WT 1a (Table 1 and Fig 1C). These mutants were comparable to that of a helix A mutant, L3A, which has 3 leucine residues replaced by alanine in helix A, is not associated with the nER membrane, and is defective in supporting the viral replication (Fig 1C) [20]. Only 5 mutants in Group C (F423A, T425A, F427A, W428A, and S433A) produced (-)RNA3 that were higher than 25% of WT levels. The inhibited BMV RNA replication was not due to the affected expression and/or stability by each mutation, because each mutant accumulated at similar levels to WT 1a (Table 1). These data indicated that helix B is necessary for viral replication processes.

### Helix B is necessary for the nuclear ER membrane association of BMV 1a

**Fig 2:**
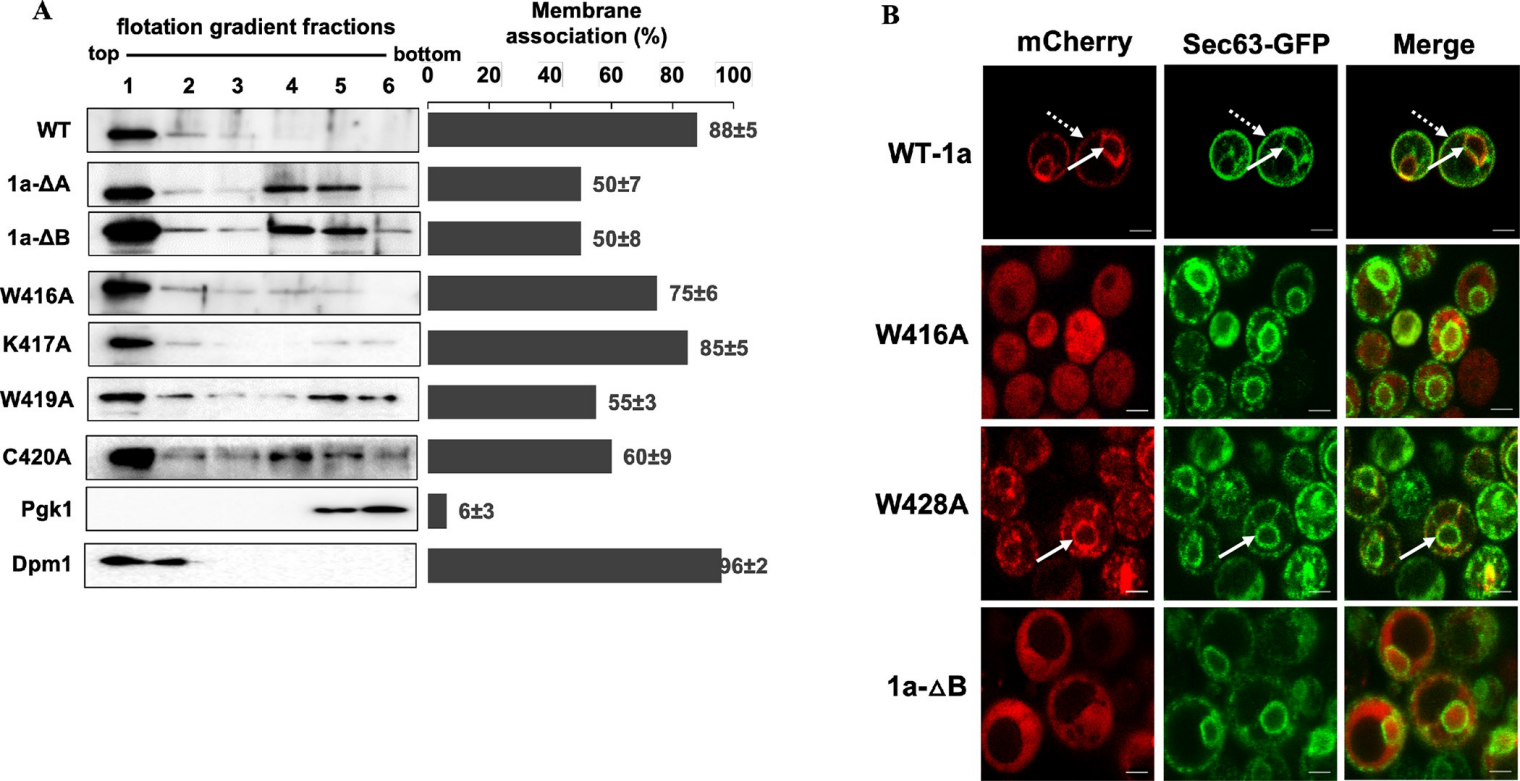
Mutations in BMV 1a helix B inhibit the nuclear ER membrane association of BMV 1a. **(A)** Membrane association of BMV 1a mutants was determined via a membrane flotation assay. Histograms represent the percentage of membrane-associated proteins as determined by the signal intensity of each protein segregated into the top three membrane fractions compared to the sum of all six fractions. Lysates of yeast cells expressing WT or mutant 1a were subjected to an iodixanol density gradient and analyzed by western blotting using antibodies against ER membrane protein Dpm1, soluble protein Pgk1, or His6 for His6-tagged BMV 1a derivatives. The values shown are from at least two independent experiments. The adjusted p-values for all samples except Dpm1 compared to WT 1a were <0.05. **(B)** Localization of mC-tagged 1a mutants in yeast cells co-expressing Sec63-GFP as examined using confocal microscopy. Nuclear ER and peripheral ER associations are indicated by solid and dotted arrows, respectively. The yellow signal in the merge panels is representative of colocalization of mC-tagged WT or mutant 1a with Sec63-GFP. Scale bars: 2μm.

To assess the impact of each mutation on 1a’s membrane association, we performed a membrane flotation assay by an iodixanol density gradient. Membrane flotation assays distinguish membrane-associated or -integral proteins from those that are soluble or cytoplasmic. Using the established conditions where membranes and membrane-proteins float atop the gradient, and soluble fractions sink to the bottom [19], we confirmed that WT 1a and membrane-protein dolichol-phosphate mannosyltransferase (Dpm1) were both detected primarily in the top two fractions, and the soluble protein phosphoglycerate kinase (Pgk1) was in the bottom two fractions (Fig 2A). Membrane association was determined as the percentage of a protein in the top three fractions against the sum of all 6 fractions (Fig 2A). About 88% of WT 1a, 96% of Dpm1, and 6% of Pgk1 were detected in the top 3 fractions (Fig 2A).

We chose five replication defective mutants (Fig 1C) for the membrane flotation analysis, including 1a-ΔB with helix B deleted, W416A, W419A, C420A, and K417A. The mutant W416A, W419A, C420A form the predicted hydrophobic face of the amphipathic helix (Fig 1B) and are also present at the beginning of the helix sequence. The mutant K417A was chosen due to its charged nature and potential contribution to membrane association. The 1a-ΔB mutant showed a 50% accumulation in the membrane fractions. The other 50% accumulated in the cytosolic fractions, with most accumulated in fractions 4 and 5, similar to the previously characterized deletion mutant 1a-ΔA [20]. Mutants W416A and K417A showed a strong membrane association with 75% and 85% of total protein in the membrane fractions, whereas W419A and C420A showed membrane association of about 55% and 60%, respectively (Fig 2A).

Since membrane flotation results are not informative in terms of specific organelle membranes where target proteins reside, we checked the localization for all 1a mutants using fluorescence and/or confocal microscopy. Since 1a predominantly localizes to the nER membrane when expressed alone [25], we expressed 1a helix B mutants with a C-terminally fused mCherry (mC) in yeast cells along with an ER protein marker, Sec63, with a C-terminally fused GFP. Since proteins could obtain distinct conformation due to the presence of an additional protein moiety [26,27], we incorporated a 32 amino acid linker (herein L32) [28] with desirable small polar amino acids, such as Thr, Ser, and Gly, between the carboxy-terminal of the protein and mC. The mC-tagged WT 1a protein was efficiently localized to the nER (the inner ring pointed by solid arrows in Fig 2B) and the peripheral ER regions (the outer ring as pointed by dotted arrows). The linker region by itself lacked the capacity to target mC to either nER or the peripheral ER regions (S1A Fig). When helix B was deleted from 1a, the mC signal was throughout the cytoplasm, indicating that helix B was necessary for 1a’s association with the nER membrane (Fig 2B). Helix B mutants with single substitutions that were severely defective in replication were segregated into two sets based on their ability to localize to the nER membrane, as shown for a representative mutant for each set in Fig 2B. Mutants in Group A (W416A, G418A, W419A, C420A, H422A, K424A, R426A, and G431) were detected mostly throughout the cell (Fig 2B and Table 1). Group B comprised of mutants that could partially localize to the nER membrane, including K417A, H421A, W429A, G430A, and D432A (Table 1). Although Group B mutants retained their ability to associate with the nER membrane, there were not as many cells showing nER localization as observed in cells expressing WT 1a protein (87%, Table 1). For Group C mutants that supported >25% of viral RNA synthesis, they behaved like those in Group B in that they colocalized with Sec63-GFP in a decreased percentage of cells compared to that by WT 1a (Table 1). We also noticed puncta in mutants F423A, T425A, and W429A (S1B Fig).

### Helix B is sufficient to target soluble proteins to the nER membrane

It was previously reported that helix A alone or the entire region E is capable of targeting GFP to the membrane fractions based on membrane flotation assays [20]. To estimate the membrane association capacity of helix B in comparison to the entire region E or helix A, we performed a membrane flotation assay using lysates from cells expressing GFP-tagged region E, helix A, helix B, or helices A and B.

**Fig 3:**
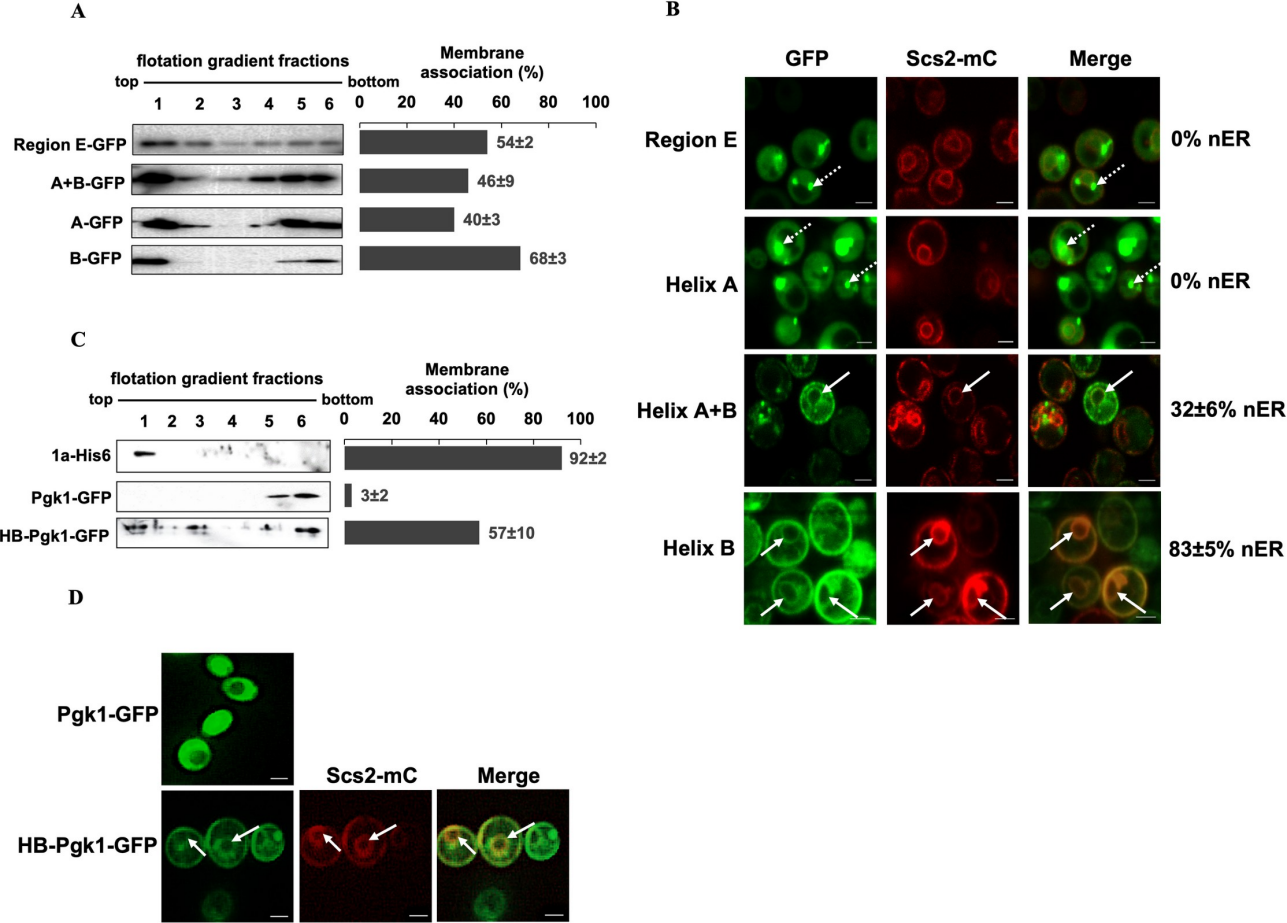
Helix B is capable of targeting soluble proteins to the nuclear ER membrane. **(A)** Membrane flotation analysis of GFP-fused region E, helices A+B, helix A, and helix B. Histograms represent the percentage of proteins detected in the top three fractions compared to the sum of all six fractions. A representative western blot is shown, with bands corresponding to respective 1a fragments detected using anti-GFP antibody. The values indicated were obtained from at least two independent experiments. **(B)** Localization of GFP-tagged region E, helices A+B, helix A, or helix B co-expressed with an mC-tagged ER marker protein Scs2. Dotted arrows point to punctate structures and solid arrows point to the nER membrane. Images were obtained using a confocal microscope. **(C)** Membrane flotation analysis for Pgk1-GFP and HB-Pgk1-GFP, using 1a-His6 as a positive control. The values are indicative of two independent experiments. **(D)** Colocalization of HB-PGK-GFP at the nER membrane with Scs2-mC. Solid arrows point to the nER membrane. Images are obtained using a fluorescence microscope. Scale bars: 2μm.

Consistent with previous studies, when fused to region E or helix A, 54% and 40% of GFP were detected in the membrane fractions compared to the sum of GFP signals across all fractions, respectively [19,20]. Cells expressing both helices showed 46% of GFP in the membrane fractions but GFP was detected across all the collected fractions, similar to cells expressing region E-GFP. Surprisingly, 68% of helix B-GFP was associated with the membrane fractions and more importantly, helix B-GFP was primarily detected in fraction 1, suggesting that helix B may play an important role in targeting 1a to membranes (Fig 3A).

Since membrane fractions represent many cellular organelle membranes, we turned to microscopy to determine whether helix B-GFP is associated with the nER membrane specifically. To assess whether helix B-GFP is associated with the nER membrane, we expressed helix B-GFP along with an mC-tagged ER marker Scs2. We also included helix A and region E for comparative analysis. Each was expressed under the control of the *GAL1* promoter, and the cells were harvested across various time points post induction when the carbon source was switched from raffinose to galactose. Region E did not localize GFP to the nER membrane across various time points but aggregated as puncta in cells (Fig 3B), consistent with the previous report [19]. These punctate structures that were observed at 6–7 hours post-induction became more diffused into the cytosolic region at 12 hours post-induction. Helix A-GFP primarily showed a rather peripheral and dispersed localization along with strong puncta (Fig 3B). Cells expressing a 1a fragment encompassing both helices A and B showed an association with nER and peripheral ER membranes in ~32% of the cells (n = 150) where both GFP and mC signals were present. Helix B-GFP, agreeing well with the membrane flotation results (Fig 3A), was detected as a two-ring pattern typical of nER and peripheral ER association in nearly 83% of the cells (n = 150, Fig 3B).

Besides GFP, helix B was sufficient to target mC to the nER membrane, similar to full-length 1a (S1A Fig). Shorter helix B sequences spanning only the first seven (W416-H422) or eleven (W416-R426) amino acids of helix B failed to target mC to the nER (S1C Fig), suggesting that the amphipathicity is governed by more than the first eleven residues of helix B. Even though a 32 amino acid-long linker was inserted between helix B and GFP/mC in various constructs, the linker was not necessary as helix B-fused mC without the linker also colocalized with Sec63-GFP at the nER membrane (S1A Fig).

To further determine the capacity of helix B in targeting other soluble proteins to the nER membrane, we fused helix B at the N-terminus of an endogenous yeast cytosolic protein. We chose yeast Pgk1, a soluble protein that is primarily detected in the cytoplasm when fused with GFP (Fig 3D) and readily detected at the bottom-most fractions in our membrane flotation analysis in the absence (Fig 2A) or presence of GFP (Fig 3C). The fusion protein, HB-Pgk1-GFP gained the ability to associate with membranes and 57% of protein was detected at the top three fractions (Fig 3C). In agreement with the membrane flotation results, GFP signals also localized to the nER and peripheral ER regions, colocalizing with Scs2-mC (Fig 3D), similar to WT 1a (Fig 2B). Collectively, our results indicated that Helix B acted as a targeting peptide that directed soluble proteins to the nER membrane.

### The amphipathic α-helix represented by helix B is functionally conserved among viruses in the *Alsuviricetes* class

BMV is a representative member of the alphavirus-like superfamily, which includes numerous viruses in the *Alsuviricetes* class. Viruses in this superfamily contain a methyltransferase-guanylyltransferase (MTase-GTase) domain whose activity has been experimentally confirmed in the nsP1 of SFV and Sindbis virus [29,30], 1a of BMV [16,17], and open reading frame 1 (ORF1) of hepatitis E virus (HEV) [31]. Among the *Alsuviricetes* class, a conserved “Iceberg” region has been identified at the C-terminus of the viral MTase-GTase domains with the presence of two amphipathic helices [21], similar to the helices A and B of 1a.

Since amphipathic helix B is a predicted secondary structure conserved across the *Alsuviricetes* class [21] with the actual predicted boundary of each helix varying across the class, we wanted to test whether the predicted helix B from other members is also functionally conserved in terms of targeting the replication protein to specific organelle membranes. We noticed that the primary sequence conservation is retained for members in the same genus, such as BMV and CCMV [32], whereas the conservation is not high across members of a family, including BMV and CMV, where BMV is a type member of the genus Bromovirus and CMV is of the genus Cucumovirus (S2A Fig). The primary sequence conservation of helix B was even lower for human viruses such as HEV and RuV when compared against BMV helix B (S2B Fig) [21].

**Fig 4:**
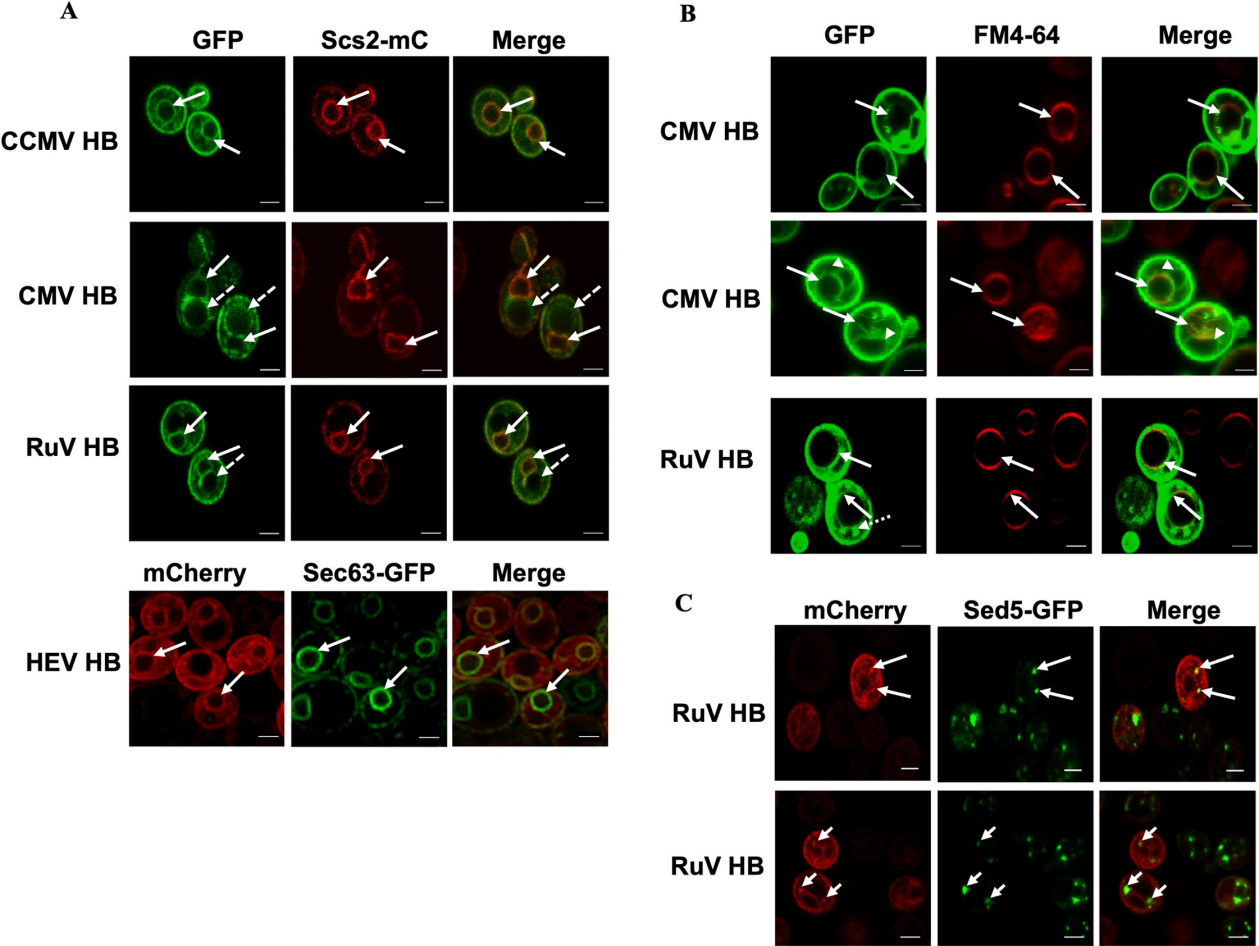
Helix B-mediated organelle targeting is conserved across members of the *Alsuviricetes* class. **(A)** Colocalization of GFP-tagged HB of CCMV, CMV, or RuV with an ER marker Scs2-mC, and colocalization of the mC-tagged HEV HB and Sec63-GFP at the nER membrane. Solid arrows point to the nER membrane and dotted arrows point to the putative vacuole membrane. **(B)** Colocalization observed for CMV HB and RuV HB with vacuole membranes stained by a lipophilic dye, FM4-64 [34]. The solid arrows point to vacuolar membranes, the arrowheads point to the putative nER membrane, and the dotted arrow in the RuV-HB panel shows punctate structures. **(C)** Colocalization of RuV HB-mC with GFP-tagged Sed5, a marker for vesicles that traffic from the ER to the Golgi. Images were obtained using a confocal microscope. Scale bars: 2μm.

We fused the predicted helix B of CCMV, CMV, HEV, and RuV to the N-terminus of fluorescent proteins and tested the localization of each recombinant protein in yeast cells, along with an ER marker, Scs2-mC or Sec63-GFP. The CCMV helix B, when fused to GFP (CCMV-HB-GFP), was capable of targeting GFP to the nER and peripheral ER membrane and colocalized with Scs2-mC (Fig 4A). This agreed well with the previous report that CCMV replicates in the nER membrane [32,33], and CCMV 1a, when expressed in yeast cells in the absence of other viral proteins, induces the formation of spherular invaginations at the outer nER membrane [32].

Unlike BMV and CCMV, CMV replicates in the vacuole membrane of plant cells, the tonoplast [35,36]. To specifically label vacuole membranes in live yeast cells, we used the lipophilic dye, FM4-64 [34]. As shown in Fig 4B, CMV helix B targeted GFP to vacuole membranes as GFP signal was primarily colocalized with the FM4-64-stained membranes. At 8–9 hours post induction of its expression, CMV-HB-GFP was found to be colocalized with the FM4-64-stained vacuole membranes in 83% of cells (n = 243), confirming that helix B of CMV 1a plays an important role in targeting CMV 1a and establishing VRCs at tonoplasts [35,36]. However, among these 83% cells, we have found two sets of localization patterns: in 42% of cells, CMV-HB-GFP was primarily associated with vacuole membranes, and peripheral ER membranes (Fig 4B, top panel). In the other 41% of cells, CMV-HB-GFP was not only associated with vacuole and peripheral ER membranes but also possibly the nER membrane (Fig 4B, middle panel). In addition, in 17% of cells, CMV-HB-GFP was only at the putative nER membrane. We further confirmed the putative nER membrane by coexpressing CMV-HB-GFP and Scs2-mC. As shown in Fig 4A, two rings were found inside the cells: the smaller ring was labeled by Scs2-mC, and the larger ring (pointed by dashed arrow) was putative vacuole membrane. It is possible that CMV HB-GFP was localized first to ER membranes, following the protein secretory pathway, before reaching vacuole membranes.

HEV is a non-enveloped (+)RNA virus in the family of *Hepeviridae* [37]. It is one of the leading viral pathogens causing acute hepatitis. HEV encodes 3 major ORFs. ORF1 is a polypeptide that is processed by viral encoded proteases. Two amphipathic alpha-helices, helix A (aa 362–380) and B (aa 399–417), are also present in the Y domain (aa 216–442) of ORF1. When fused to mC, Helix B of HEV ORF1 also colocalized with the GFP-Sec63 at the nER membrane (Fig 4A), which is the purported site of HEV replication [37]. However, mC signal was also found between the nER and peripheral ER, similar to tubular ERs that connect the nER and peripheral ER.

RuV is known to utilize multiple organelle membrane sites for its replication. These membranes include those of Golgi, endosomes, vesicles, and vacuoles of endolysosomal origin in addition to ER [38]. The viral protein P150 is sufficient for organelle targeting [39] and also contains a Y domain similar to HEV with the presence of two amphipathic helices. We expressed RuV helix B with a C-terminally fused GFP and confirmed its localization at the vacuolar regions using FM4-64 that stains the vacuolar membrane (Fig 4B), and its localization at the nER by using the ER marker Scs2-mC (Fig 4A). We also fused RuV HB (aa 423–434) to mC and coexpressed RuV-HB-mC along with an early Golgi compartment marker Sed5 that was fused with GFP. As shown in Fig 4C, RuV-HB-mC did colocalize with the punctate structures seen throughout the cytoplasm labeled by Sed5-GFP.

### Helix B motifs from BMV and CMV target fluorescent proteins to ER membranes or tonoplast in plant cells

**Fig 5:**
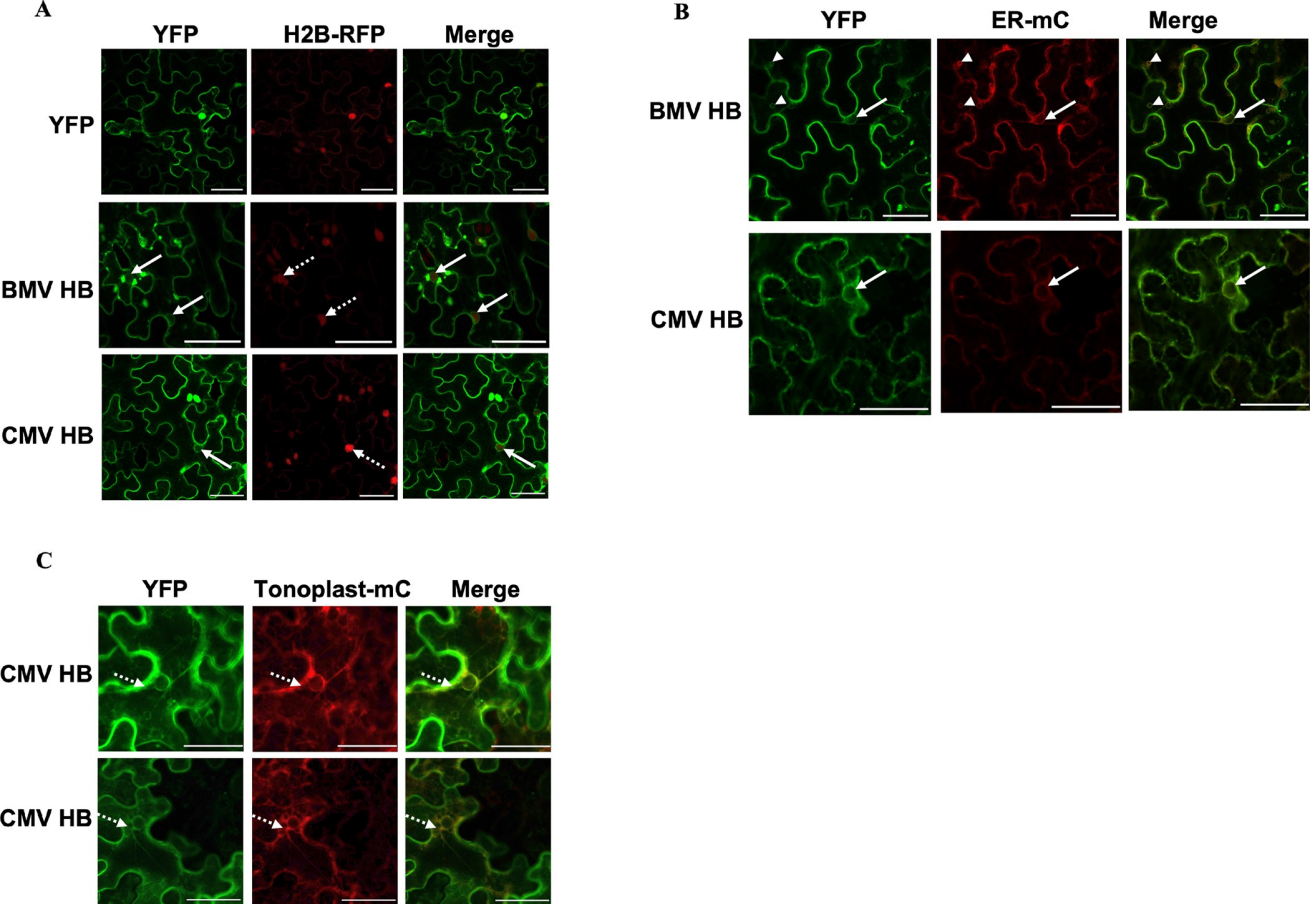
Localizations of BMV and CMV helix B in *Nicotiana benthamiana* cells parallel those in yeast cells. **(A)** YFP-tagged helix B from BMV or CMV was expressed in histone 2B-RFP transgenic *N*. *benthamiana* plants via agroinfiltration. Solid and dotted arrows point to the nER membrane and the nucleus, respectively. **(B)** BMV or CMV HB-YFP was co-expressed with an mC-tagged ER marker. Arrows point to the nER membrane and arrowheads point to small vesicles that are colocalized with the ER marker. **(C)** Colocalization of CMV HB-YFP with an mC-tagged tonoplast marker as indicated by dotted arrows. The fluorescence signal was observed under laser confocal microscopy at 40 hpai. Scale bars: 50μm.

The ability of BMV and CMV helix B to target GFP to the nER and/or vacuole membranes in yeast cells prompted us to check whether these helices could do the same *in planta*. To test this, we fused BMV or CMV helix B to the N-terminus of YFP in a T-DNA binary vector under the control of the 35S promoter of cauliflower mosaic virus (CaMV) [40] and expressed by agroinfiltration in histone 2B-RFP (H2B-RFP) transgenic *Nicotiana benthamiana* plants [41] (Fig 5A), or *N*. *benthamiana* plants expressing either ER (Fig 5B) or tonoplast (Fig 5C) markers. The localization of YFP signal in *N*. *benthamiana* cells was observed under a laser confocal microscope at 40 hours post agroinfiltration (hpai), which is the commonly used time point to observe transiently expressed proteins [40]. While free YFP was observed in the cytoplasm and the nucleus, BMV HB-YFP signal was observed surrounding the histone 2B-RFP-represented nucleus (Fig 5A). To further confirm BMV HB-YFP was associated with ER membranes, we co-expressed BMV HB-YFP and an mC-tagged ER marker [42]. The YFP signal was colocalized with the mC signal in the nER and peripheral ER membranes (Fig 5B). In addition, we have observed multiple ER-localized aggregates that were not present in cells expressing YFP (Fig 5A). Surprisingly, we also observed small vesicle-like structures that contain both signals of YFP and mC (arrowheads in Fig 5B), indicating the ER origin of these vesicles. However, the possible functions of these vesicle-like structures are unclear.

We next tested the localization of YFP-tagged CMV HB in H2B-RFP-transgenic *N*. *benthamiana* cells. Similar to what we have observed in yeast cells, CMV HB-YFP was localized at ER membranes surrounding the nucleus and colocalized with the ER marker (Fig 5A and 5B). Agreeing well with the report that CMV 1a, when expressed alone, localizes to vacuolar membranes [36], CMV HB-RFP was detected primarily at the tonoplasts as it colocalized with an mC-tagged tonoplast marker (Fig 5C). These results confirmed the findings observed in yeast that CMV HB can target YFP to both ER and vacuole membranes. We also included YFP-tagged CCMV HB in our study and found the nER localization in these cells (S3 Fig), agreeing well with the results in yeast cells (Fig 4A).

## Discussion

The membrane association of viral replication complexes is a key feature across all (+)RNA viruses. Different (+)RNA viruses replicate in specific organelle membranes. BMV, CCMV, and beet black scotch virus replicate in ER membranes, whereas CMV, tomato bushy stunt virus, barley stripe mosaic virus, and turnip crinkle virus prefer membranes of the tonoplast, peroxisome, chloroplast, and mitochondria, respectively [33,35,43–47]. Some plausible reasons for the varied preference of membranes are believed to be due to the membrane lipids and/or protein compositions [48–52]. However, it is still unclear what structural determinant(s) within the viral replication proteins that are responsible for targeting these proteins to specific organelles, especially for those that lack TMDs, such as BMV 1a. We report here that a single amphipathic α-helix in 1a, helix B, plays a key role in determining 1a’s association with the nER membrane. Helix B is necessary for 1a’s nER membrane association and is sufficient to target several soluble proteins to the nER membrane, extending our understanding beyond its role as to regulate the oxidizing potential of the ER lumen [22]. We further show that a similar helix in the replication proteins of CCMV, CMV, HEV, and RuV is able to target fluorescent proteins to the specific organelles where their VRCs are formed, revealing a conserved feature among members of the *Alsuviricetes* class.

### BMV 1a helix B is necessary and sufficient for 1a’s association with the nuclear ER membrane

Many viral replication proteins from (+)RNA viruses contain TMDs that target them to the designated organelle membranes for initiating the VRC formation. Protein A of FHV, when expressed alone, is targeted to the outer mitochondrial membrane where FHV forms VRCs [8,53]. Protein A has an N-terminal TMD (aa 1–46) that is sufficient to target GFP to mitochondrial membranes [8]. Replication proteins of some (+)RNA viruses lack a TMD but possess amphipathic helix or helices, includes BMV 1a (helix A, aa 392–407 and helix B aa 416–433) [20–22], poliovirus 2C (aa 19–36) [10,54], Hepatitis C virus 5A (aa 7–28) [55], and SFV nsP1 (aa 245–264) [56], among others.

Amphipathic helices have long been recognized as generators and/or sensors of membrane curvatures [24,57]. Residues on the hydrophobic face of the helix insert into the fatty acid tails while polar residues are involved in electrostatic interactions with the polar head of phospholipids [58]. It has long been understood that the amphipathic helix serves as an anchor, inserting TMD-lacking viral proteins shallowly into the membranes. Our recent data indicated that the helix of poliovirus 2C played an active role in remodeling membranes [59]. In an *in vitro* tubulation assay, the synthesized peptide representing the amphipathic helix of poliovirus 2C converted large spherical liposomes into tubules and smaller vesicles that were stable for a week [59]. In addition, mutations disrupting the helicity of the 2C helix inhibited membrane remodeling *in vitro* as a synthesized peptide and blocked viral replication when incorporated into the full-length viral genome [59].

Helix B of BMV 1a was first predicted by Ahola and Karlin while analyzing BMV 1a, CMV 1a, and equivalent replication proteins among the *Alsuviricetes* class [21]. The predicted helix B was further characterized for its amphipathicity and its viroporin activity that allows oxidizing environment within BMV VRCs [22]. In this study, we determined that helix B is necessary and sufficient for BMV 1a’s association with nER membrane, and thus, essential for BMV genomic replication (Table 1). The vast majority of the helix B mutants (group A and B) with an Ala substitution either failed or supported no more than 10% of BMV genomic replication (Table 1), indicating a crucial role for helix B in this process. This also aligns with the reported helix B mutants K424G and R426K for selectively lacking viroporin activity that failed to support BMV genome replication [22].

The 19 helix B mutants generated in this study can be divided into three groups based on genomic replication and their localization. Group A contains nine mutants, including the helix B deletion mutant and eight Ala-replacement mutants, which were primarily detected in the cytoplasm and possibly peripheral ER membranes (Table 1). Future experiments will further address to which organelle(s), other than the nER, these 1a mutants were targeted. Group B includes five mutants that were associated with the nER at lower efficiency than WT, ranging from 17% to 36% of cells (Table 1). For instance, K417A was detected in the nER membrane in 36% of cells, compared to 87% of cells with WT 1a (Table 1). Mutants in both Groups A and B supported less than 10% of viral genomic replication compared to WT 1a. Another five mutants that supported partial replication and were inefficiently localized at the nER membrane are assigned to Group C. It is possible that some mutants were only transiently associated with the nER. Given the fact that these mutants accumulated at similar levels to WT (Table 1), we concluded that helix B is necessary for BMV 1a’s nER membrane association and thus, genome replication.

Soluble proteins, GFP (Fig 3B), mC (S1A Fig), and Pgk1 (Fig 3D), were targeted primarily to the nER and also peripheral ER membranes when fused to helix B at their N-terminus. However, helix A-tagged GFP did not (Fig 3B), indicating that BMV 1a helix B plays an important role in 1a’s targeting to the nER membrane. We are unable to determine whether helix B-mediated nER targeting is due to the specific lipid composition of the organelle membranes or whether it interacts with a specific host protein that localizes to the nER [60]. It will be exciting to study in the future whether targeting is an active process requiring host machinery in trans or whether it is a passive process relying on cis-elements within the helix and their internal propensity to bind the lipid bilayer.

### Helix B-mediated organelle targeting is a conserved feature across members within the *Alsuviricetes* class

The initial step for a successful viral infection is to assemble functional VRCs at the target organelle. This starts with targeting viral replication proteins to the organelle where VRCs will be established. Disruption of this step will block viral replication at its early stage. For instance, we have reported a mutant of yeast protein Cho2 (choline requiring 2), *Cho2-aia*, that retargeted BMV 1a from the nER to peripheral ER-localized puncta, and thus, significantly inhibited BMV replication [61], supporting the importance of the correctly localizing the viral replication proteins to their designated organelle membranes.

Knowing that helix B of 1a is sufficient to target soluble proteins to the site of BMV replication, we tested the conservation of this feature across the members of the *Alsuviricetes* class, given the fact that two similar amphipathic helices have been identified based on sequence analysis, with helix B immediately downstream of helix A [21]. Helix B from CCMV, which is in the same genus as BMV and replicates in the nER membrane in yeast cells [32], targeted GFP to the nER membrane as hypothesized (Fig 4A). Helix B from CMV 1a targeted GFP primarily to vacuolar membranes, similar to the tonoplast where CMV replicates in plant cells [35,36]. Importantly, the results for the helices from BMV, CMV, and CCMV were similar in both yeast and plant cells (Figs 3–5 and S3). We further demonstrated in yeast cells that predicted amphipathic helices in HEV and RuV replication proteins (S2B Fig) were able to target mC or GFP to the sites of replication, the ER membrane for HEV, and Golgi, ER, and vacuolar membranes for RuV [37,38] (Fig 4). It should be noted that the amphipathic helices of HEV and RuV were only tested in yeast cells and subsequent validation in human cells will be further pursued in the future. Nevertheless, our work demonstrates that helix B in the replication proteins of a group of diverse viruses in the *Alsuviricetes* class is indeed a conserved determinant for targeting the viral replication proteins to their specific sites of replication.

It has been reported that the amphipathic helix (aa 7–28) in HCV 5A was able to target GFP to ER membranes, where HCV assembles its VRCs [62]. For poliovirus 2C, a 38 amino acid-long fragment (amino acid 1–38) encompassing the amphipathic helix (amino acid 19–36) was able to associate with lipid droplets, where poliovirus VRCs acquire fatty acids for VRC formation [54]. With two amphipathic helices (αA and αB) that are separated by several amino acids, the 41 amino acid-long chloroplast targeting domain (CTD) of the 140K/98K replication protein from turnip yellow mosaic virus (TYMV), another virus from the *Alsuviricetes* class, targets GFP to the outer chloroplast membranes [63]. These results have pointed out the role of amphipathic helix from individual viruses in targeting specific organelles. Our work, going further, systematically analyzed a range of helices from replication proteins of viruses in the *Alsuviricetes* class and confirmed a conserved role of these helices in targeting viral replication proteins to specific organelle membranes where VRCs are formed.

In recently solved high-resolution cryo-EM structures of nsP1 of Chikungunya virus (CHIKV), which belong to the *Alphaviridae* family along with SFV, 12 copies of nsP1 assembled as a crown-shaped ring with a C12 symmetry in association with the bilayer membrane-mimicking detergent micelles [64,65]. The two membrane interacting loops form amphipathic spikes that penetrate into the micelle, with a hydrophobic tip and a positively charged belt. Although the helix of SFV nsP1 (aa 245–264) has been well documented as inserted into membranes [56], the equivalent helix in CHIKV (aa 244–263) was not in the membrane-binding spike [64,65]. It is possible that the helix makes initial contact with the membrane, but as the proteins oligomerize, significant conformational changes occur so that the helix becomes part of the protein core and it no longer contacts the membrane. It is of great interest to determine if helix A and/or helix B of BMV 1a or other replication proteins from the *Alsuviricetes* class are embedded within the membrane or if they behave similarly to CHIKV nsP1.

### Different BMV 1a helices may play different roles in the VRC formation

The helix A peptide of 1a becomes helical when incubated with lipid bilayer-mimicking micelles [20]. Mutations in helix A generated two groups of mutants that were all defective in supporting BMV genome replication. Group I mutants did not associate with the nER membrane, indicating a necessary role of helix A in 1a’s nER association. Mutants in group II induced the formation of VRCs that were more abundant but smaller in size compared to those induced by WT 1a, pointing out a possible role in generating or facilitating the remodeling of membranes into VRCs [20]. Helix A was able to target GFP to membrane fractions in the membrane flotation assay [20], however, our data showed that helix A was not able to target proteins to the nER membrane on its own (Fig 3B). Further, helix A and B helices together were not as effective as helix B alone in targeting GFP to the nER membrane (Fig 3B). It is possible that the presence of helix A might affect the conformation of helix B and additionally, the context in which helix B is positioned is also critical because the 1a-ΔA mutant, which contains helix B but not helix A [20], was not associated with the nER membrane. In this work, each helix B from viral replication proteins was fused to the N-terminus of fluorescence proteins. The effect of the position in which helix B is fused to soluble proteins on the localization of these proteins will be further pursued in the future. Nevertheless, based on the data from Liu et al [20] and our results, we propose that helix A may participate in membrane remodeling but not 1a targeting, and helix B plays a key role in targeting 1a to the nER membrane.

In this regard, the roles of BMV 1a helix A and helix B are different from αA and αB in the CTD of TYMV 140K/98K replication protein. TYMV is in the Tymo group of the alphavirus-like superfamily, different from the Alto group that includes BMV, CCMV, CMV, HEV, and RuV [21]. TYMV replicates at the outer membrane of chloroplasts and its replication protein 140K/98K is responsible for targeting and remodeling the chloroplast membranes [63], similar to the roles of BMV 1a. The CTD is able to target GFP to the chloroplast membranes. However, it has been clearly demonstrated that disrupting the helicity of either αA or αB does not affect the CTD’s ability to target GFP to the chloroplast membrane, indicating either helix is sufficient when present in the CTD. This is in contrast to the BMV 1a helices A+B domain that showed much reduced efficiency in targeting GFP to the membrane fraction (Fig 3A) and the nER membrane (Fig 3B) compared to those of 1a helix B alone. It should be also noted that it is unclear whether αA or αB is sufficient to target GFP to chloroplast membranes when either of them is fused to GFP independent of the CTD.

### Possible mechanisms governing the amphipathic helix-mediated membrane targeting

It is not yet clear how each replication protein is targeted to a specific organelle membrane. There are at least two potential nonexclusive mechanisms by which viral proteins target specific membranes: the viral proteins associate with specific lipids enriched on those membranes, or there are host proteins on those membranes that act as receptors for the viral proteins. For cellular amphipathic helices that have been extensively studied, some can sense and bind specific lipids, and some are involved in inducing curvatures [66]. For instance, the amphipathic helix in yeast transcription repressor Opi1 (overproduction of inositol 1) binds preferentially to phosphatidic acid (PA) over phosphatidylserine (PS) *in vitro* [67]. On the contrary, the amphipathic helix in yeast protein Spo20 (sporulation 20) prefers charged lipids, and binds PA and PS with similar affinity [68]. Given that organelle membranes have different lipid compositions [7,69], it is possible that amphipathic helices from BMV 1a, CCMV 1a, and HEV ORF1 may recognize certain lipids present in ER membranes while helix B in CMV 1a and RuV 1a prefer lipids enriched in the vacuole membranes. In the future, the binding properties of these peptides to different organelle membranes can be examined by using *in vitro* assays [59] with liposomes composed of different lipids.

We showed that CMV HB-GFP were at both ER and vacuolar membranes (Fig 4A and 4B), suggesting that CMV HB-GFP was likely transported to ER membranes first before reaching the vacuolar membrane via the cellular protein secretory pathway. Of note, we have previously found that several components of the cellular COPII (coat protein complex II) pathway were required for BMV 1a’s association with the nER membrane [60], supporting our idea that the protein secretory and protein trafficking pathway is needed for these helix-mediated protein targeting.

In conclusion, we have demonstrated that helix B of BMV 1a is necessary and sufficient for 1a’s targeting to the nER membrane, and thus, plays a critical role in initiating BMV VRC formation at the site of viral replication. The conclusion that an amphipathic helix dictates the targeting of a viral replication protein can be extended to several viruses belonging to the *Alsuviricetes* class, revealing a conserved feature among a group of viruses infecting plants, animals, and humans. Given that targeting viral replication proteins to the designated organelle is the initial step to form functional VRCs for genomic replication, a better understanding will provide opportunities to develop antiviral strategies for virus control.

## Materials and Methods

### Yeast, Plant, and Growth conditions

The *Saccharomyces cerevisiae* strain YPH500 (*MAT*α *ura3–52*, *lys2–801*, *ade2–101*, *trp1*-Δ*63*, *his3*-Δ*200*, *leu2*-Δ*1*) was used in all experiments. Yeast cells were grown at 30°C in a synthetic complete medium containing 2% raffinose or galactose as the carbon source. Histidine, tryptophan, uracil, leucine, or combinations of them were omitted from the medium to maintain selection for different plasmid combinations. For cells used to analyze viral replication, cells were harvested as described previously [61] when the optical density at 600 nm (OD_600_) reached between 0.6 and 1.0.

All agroinfiltration experiments were performed in WT or H2B-RFP (red fluorescent protein fused to the C-terminus of histone 2B) transgenic *N*. *benthamiana* plants. Plants were grown in a growth chamber with temperatures at 26°C (16 h, light) or 22°C (8 h, dark) for 4–6 weeks before being infiltrated with agrobacterium cultures [41]. After infiltration, the plants were kept under the same growth conditions.

### Plasmids

To launch BMV replication, BMV 1a or mutant derivatives, 2a^Pol^ and RNA3 were expressed under the control of the *GAL1* promoter from centromeric plasmids pB1YT3-cH6 (with a His6 tag at the C-terminus of BMV 1a or derivatives), pB2YT5, and pB3MS82, respectively [17]. pB3MS82 is to express an RNA3 derivative, in which the coat protein gene has a four-nucleotide insertion and thus, no coat protein is produced [70]. pB1YT3-mC [61] was used as a vector to incorporate a 32 amino acids linker (-DVGGGGSEGGGSGGPGSGGEGSAGGGSAGGGS-) between BMV 1a and mC. pB1YT3-L32-mC or pB1YT3-L32-GFP was used as the vector to introduce mutations in helix B by an overlapping PCR approach using specific primers with mutations incorporated [71]. The construct pB1YT3-L32-GFP was made by replacing mC with GFP to express GFP-tagged WT 1a. BMV 1a was replaced with BMV region E, helix A+B, helix A, or helix B to make constructs pE-, pHAB-, pHA-, or pHB-L32-GFP or -mC.

Helix B fragments were amplified by PCR (S1 Table for primers) from cDNA of CMV 1a, CCMV 1a, HEV ORF1, and RuV P150 cloned to pB1YT3-L32-mC or -GFP by replacing the BMV 1a gene. Accession numbers of viral genome used to amplify helix B cDNA sequences are: X02380 for BMV (the Russian strain) [72], D00356 for CMV (the Fny strain) [73], M65139 for CCMV [74], JQ679014 for HEV (the Kernow C-1 strain) [75], and M15240 for RuV (the strain F-therin) [21].

Organelle markers Scs2-mC, Sec63-GFP, GFP-Sed5 were expressed under the control of an endogenous promoter from a low-copy-number plasmid.

BMV or CMV HB was amplified and inserted into the *Bam*HI-digested p1300-YFP vector [40,76] in frame with and upstream of YFP, under the control of the Cauliflower mosaic virus 35S promoter to express BMV HB-YFP and CMV HB-YFP.

### RNA extraction and northern blotting

Total RNA was isolated from harvested yeast cells by using a hot acidic phenol method [77]. Equal amounts of total RNA were analyzed by formaldehyde-agarose gel electrophoresis, followed by blotting onto Nytran nylon membranes. Specific ^32^P-labeled probes were generated by *in vitro* transcription. Radioactive signals were detected by using a Typhoon FLA 7000 phosphorimaging system and the intensity of signals was quantified using Adobe Photoshop. 18S rRNA was used as a loading control to eliminate loading variations. All RNA analysis experiments were done with at least two independent yeast transformants, which gave reproducible results.

### Protein extraction and western blotting

For protein analysis, two OD_600_ units of yeast cells were harvested in their logarithmic growth phase and frozen prior to extraction. Total proteins were extracted as described previously [60]. Briefly, yeast cells were broken in a lysis buffer (50 mM Tris-HCl pH 8.0,1 mM EGTA, 2 mM EDTA pH 8, 15 mM KCl, 10 mM MgCl2, and 20% Glycerol) containing a proteinase inhibitor mix (G biosciences) at a 1:200 dilution. SDS lysis buffer (30 mM DTT, 90 mM HEPES, pH 7.5, 2% SDS) and loading buffer was added to the mixture and boiled for 5 min, the cell debris was removed by centrifugation. Equal volumes of total lysates were analyzed using sodium dodecyl sulfate-polyacrylamide gel electrophoresis (SDS-PAGE) and transferred to a polyvinylidene difluoride (PVDF) membrane (Sigma Millipore). Rabbit anti-BMV 1a antiserum (1:10,000 dilution, a gift from Dr. Paul Ahlquist at the University of Wisconsin-Madison), mouse anti-BMV 2a^pol^ (1:3000 dilution, a gift from Dr. Paul Ahlquist), mouse anti-Pgk1 (1:10,000 dilution; Thermo Fisher Scientific), rabbit anti-GFP (1:5000 dilution, GenScript), or rabbit anti-RFP (1:3000 dilution, GenScript), mouse anti-His6 (1:3000 dilution, GenScript), mouse anti-Dpm1 (1:3,000 dilution, Thermo Fisher Scientific) was used as the primary antibody. Horseradish peroxidase-conjugated anti-rabbit or anti-mouse antibody (1:10,000 dilution, Thermo Fisher Scientific) together with the SuperSignal West Femto maximum sensitivity substrate (Thermo Fisher Scientific) was used to detect target proteins. Protein bands were detected using Azure C400 (Azure biosystems) and band intensities were quantified using Adobe Photoshop.

### Membrane flotation assays

Flotation assays were performed as described previously [48,52]. Briefly, spheroplasts prepared from ten OD_600_ units of yeast cells expressing a target protein were resuspended in 350 μl of TNE buffer (50 mM Tris-HCl [pH 8.0], 150 mM NaCl, 5 mM EDTA) containing 1:100 dilution of yeast/fungal protease arrest (G biosciences). Spheroplasts were lysed via 25 passes through a 22-gauge, 4 cm long needle. Lysates were also prepared from whole cells without spheroplasting: ten OD_600_ units of yeast cells were harvested and broken with glass beads in 350 μl of TNE buffer containing yeast/fungal protease arrest (G biosciences). The resulting cell lysates, obtained from spheroplasts or whole cells, was centrifuged for 5 min at 500 x *g* to remove cell debris. The supernatant was adjusted to 40% iodixanol by the addition of 60% iodixanol (Sigma). A 600 μl of the mixture was placed at the bottom of a Beckman TLS55 centrifuge tube and overlaid with 1.4 ml of 30% iodixanol in TNE and finally with 100 μl of TNE. The gradients were centrifuged at 201,000 x *g* at 4°C for 5 h and collected into 6 fractions. Equal volumes of the collected fractions were analyzed by SDS-PAGE and western blotting for specific proteins. The flotation data for 1a fragments were obtained using two independent experiments using whole cell lysates and once using the spheroplasts. The StDev was calculated by comparing at least two independent data sets for each flotation assay.

### Vacuolar membrane staining

To stain yeast vacuolar membranes, a lipophilic dye, FM4-64 (Thermo Fisher Scientific) that selectively stains the vacuolar membranes was used. A stock (1.6 μM in DMSO) was made and staining was performed as described previously [34]. Yeast cells at an OD_600_ of 0.6–0.8 were collected and resuspended in 50 μl YAPD, mixed with 1μl of the stock dye, and incubated in a water bath maintained at 30°C for 30 minutes. After the incubation, 1 ml YAPD was added, and the mixture was centrifuged at 5000 x *g* for 5 minutes at RT. The cell pellet was collected and resuspended in 5 ml YAPD, transferred to culture tubes, and grown for 2.5 hours. The cells were harvested by centrifugation at 5000 x *g* for 5 minutes and fluorescence was detected at an excitation/emission maximum of ~515/640 nm.

### Fluorescence and confocal imaging

For all localization studies, yeast cells co-expressing mC- and eGFP-tagged proteins were grown overnight at 30°C in a synthetic defined medium containing 2% raffinose as a carbon source. From this starter culture, a subculture with an OD_600_ of 0.3 was initiated and grown in 2% galactose as a carbon source to induce the expression of the target proteins for 7–8 hours for microscopic analysis. The GFP and mC signals were sequentially captured using a Zeiss epifluorescence microscope (Figs 3D and S1) or confocal microscope ZEISS LSM 710 (Figs 2B and 4A–4C) at an excitation of ~488 nm for GFP or 560 nm for mC.

The percentage of cells with the nuclear ER membrane localization of BMV 1a-mC/GFP was calculated based on the total number of cells that had a visible inner ring structure as observed in cells expressing fluorescently tagged ER marker protein Sec63 or Scs2. At least one hundred cells were counted for each mutant and repeated three times. The percentages depicted in the figures represent the average of all the experiments. The formula for calculating the percentage of cells with the nER localization is as follows: (number of cells with colocalized GFP and mC signals at the inner ring)/(total number of cells with visible GFP and mC signals)*100. These values were used to calculate the standard deviations across the mutants. The statistical significance was calculated by performing a Welch t-test.

For confocal microscopy in plants, the expression vectors p1300-YFP, -BMV HB-YFP, -CMV HB-YFP, and CCMV HB-YFP were individually introduced into *A*. *tumefaciens* strain GV3101. After overnight growth, Agrobacterium cultures were harvested and resuspended in the induction buffer (10 mM MgSO4, 100 mM 2-N-morpholino ethanesulfonic acid [pH 5.7], 2 mM acetosyringone), and incubated for at least 2 h at room temperature. The suspensions were then adjusted to OD600 = 1.0 and infiltrated into WT or H2B-RFP transgenic *N*. *benthamiana* plants. After agroinfiltration, *N*. *benthamiana* were grown in a growth chamber with a 16 h light/8 h dark cycle. At 40 hpai, leaves were excised and YFP fluorescence was examined in epidermal cells using confocal microscopy (ZEISS LSM 710). The microscope was configured with a 458–515 nm dichroic mirror for dual excitation and a 488-nm beam splitter to help separate YFP fluorescence. Photographic images were prepared using ZEN 2011SP1.

## Supporting information

S1 Fig The 32 amino acid-long linker is not required for the correct localization of fluorescent protein-tagged BMV 1a helix B. (PDF)

S2 Fig Two amphipathic alpha-helices are present in the replication proteins from members of the *Alsuviricetes* class. (PDF)

S3 Fig CCMV HB directs yellow fluorescent protein to ER membranes in plant cells. (PDF)

S1 Table The primers used in amplifying the Region E and helix A and/or helix B of BMV, as well as various helix B motifs of CMV 1a, CCMV 1a, HEV ORF1, and RuV P150. (PDF)
