# Supplementary material for: A conserved viral amphipathic helix governs the replication site-specific membrane association
Source: PLoS Pathog. 2022 Sep 1;18(9):e1010752. doi: 10.1371/journal.ppat.1010752 (PMC9473614; doi:10.1371/journal.ppat.1010752)
Supplement: S1 Fig — (PDF) [file ppat.1010752.s001.pdf]

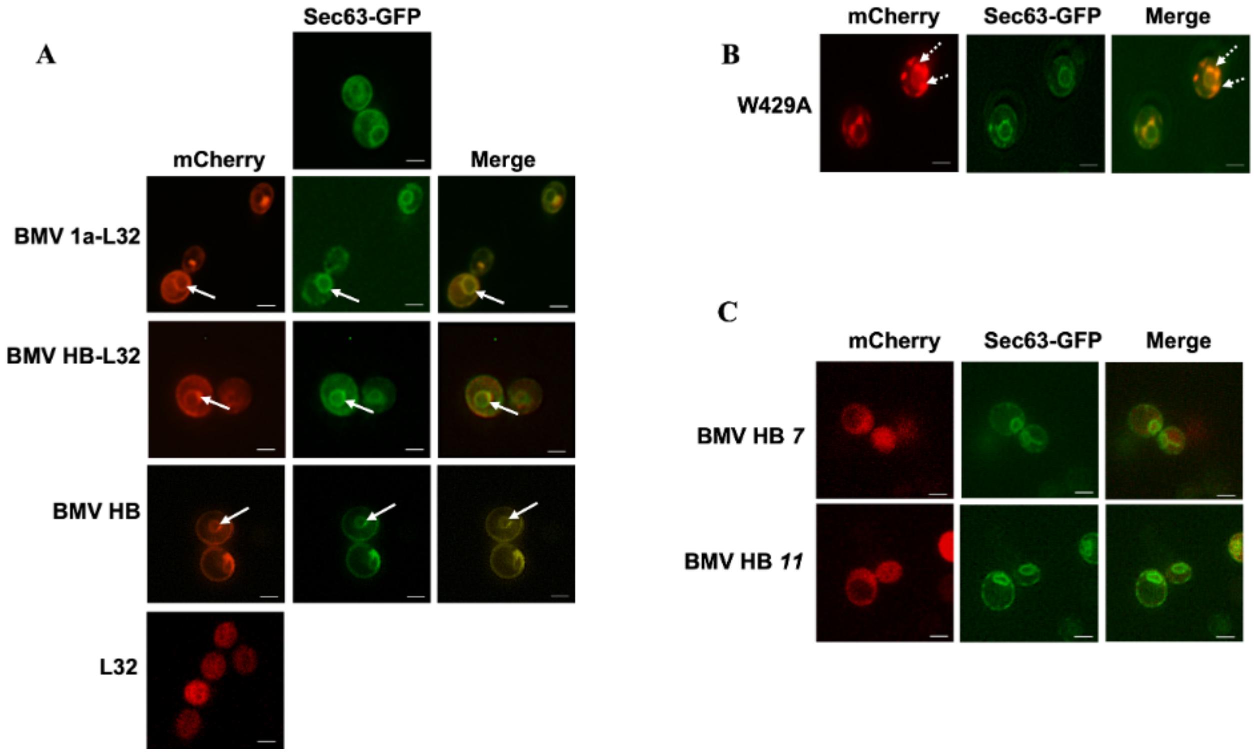

S1 Fig. The 32 amino acid-long linker is not required for the correct localization of fluorescent protein-tagged BMV 1a helix B.

(A) Fluorescence microscopic images showing that mC-tagged BMV 1a helix B with or without the 32 aa-long linker is colocalized with an ER marker Sec63-GFP. L32-mC, which is not fused to BMV 1a or helix B, is localized in the cytosol (bottom panel). **(B)** Puncta (pointed by dotted arrows) are formed in cells coexpressing Sec63-GFP and the mC-tagged 1a mutant W429A. **(C)** Truncated versions of helix B spanning amino acids 416-422 (BMV HB *7*) or 416-426 (BMV HB *11*) fail to target mC to ER membranes. Scale bars: 2µm.
