# Supplementary material for: A conserved viral amphipathic helix governs the replication site-specific membrane association
Source: PLoS Pathog. 2022 Sep 1;18(9):e1010752. doi: 10.1371/journal.ppat.1010752 (PMC9473614; doi:10.1371/journal.ppat.1010752)
Supplement: S2 Fig — (PDF) [file ppat.1010752.s002.pdf]

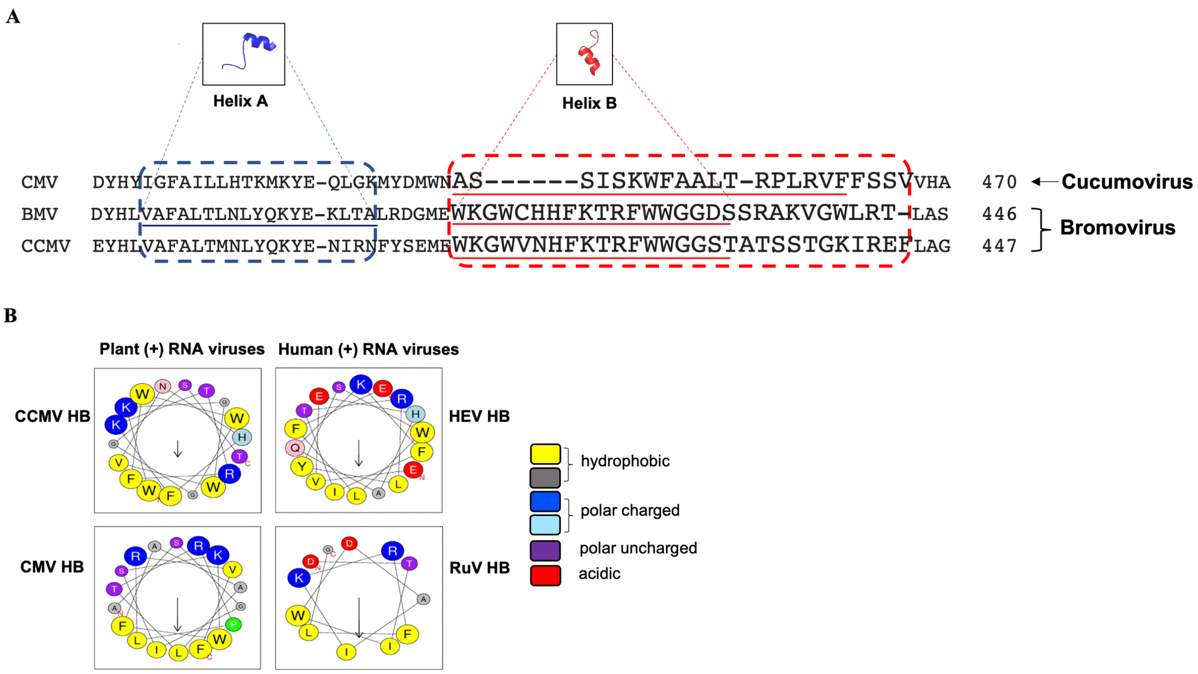

Virus name	The protein harboring helix B	Position of helix B in the protein	Sequence
HEV	ORF1	399-418 aa	EVEHAQKFITRLYSWLFE
RuV	p150 protease	423-434 aa	DTIWDAIKRFLG

S2 Fig. Two amphipathic alpha-helices are present in the replication proteins from members of the *Alsuviricetes* class.

(A) Multiple sequence alignment shown for a short stretch of region encompassing helix A and helix B across plant viruses, BMV, CCMV, and CMV. The members of a genus share higher identity in helix A and helix B (CCMV and BMV) than members from higher ranks such as family (BMV and CMV). **(B)** Helical wheel projections of amphipathic helix B of plant (CMV, CCMV) and animal viruses (HEV, RuV) using the predicted helix B regions. Amphipathic faces for CCMV and CMV as predicted by HeliQuest (https://heliquest.ipmc.cnrs.fr/) are WGFWFV and PWFLILFA, and for HEV and RuV are LALIVY and AFIILW, respectively. The arrow in helical wheels corresponds to the hydrophobic moment. The table shows the helix B sequences of HEV and RuV.
