# Supplementary material for: A conserved viral amphipathic helix governs the replication site-specific membrane association
Source: PLoS Pathog. 2022 Sep 1;18(9):e1010752. doi: 10.1371/journal.ppat.1010752 (PMC9473614; doi:10.1371/journal.ppat.1010752)
Supplement: S3 Fig — (PDF) [file ppat.1010752.s003.pdf]

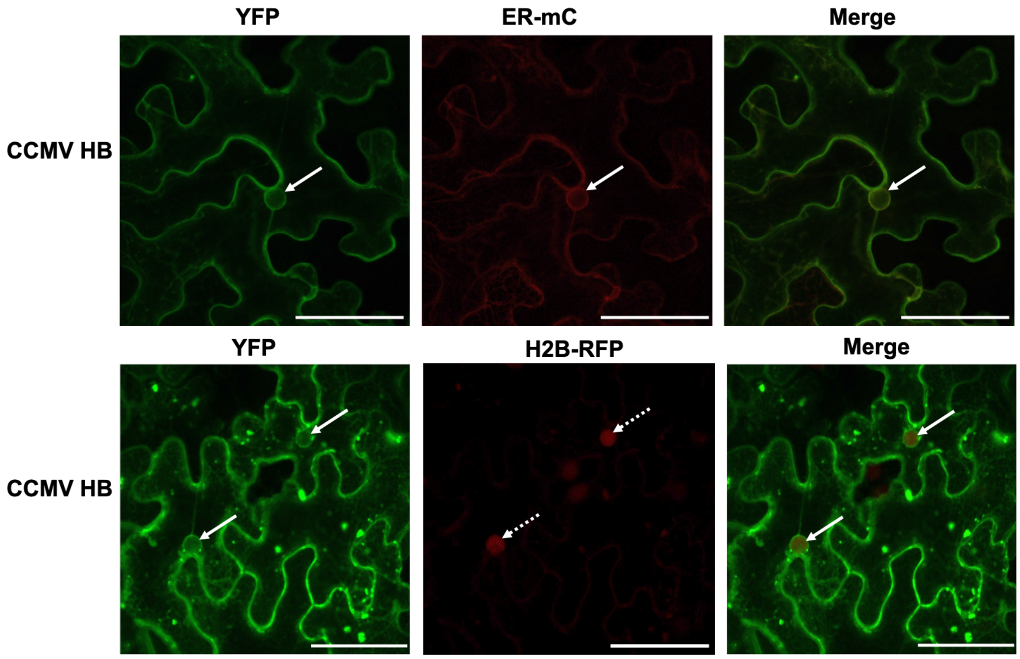

S3 Fig. CCMV helix B directs yellow fluorescent protein to ER membranes in plant cells.

Confocal microscope images showing YFP-tagged CCMV 1a helix B (HB) with an mC-tagged ER marker (top row). The bottom panel shows the localization of YFP-tagged CCMV 1a HB in histone 2B-RFP transgenic *N. benthamiana* cells. Solid arrows denote the nER localization and dotted arrows represent the nucleus. Scale bars: 50µm
