# Supplementary material for: A conserved viral amphipathic helix governs the replication site-specific membrane association
Source: PLoS Pathog. 2022 Sep 1;18(9):e1010752. doi: 10.1371/journal.ppat.1010752 (PMC9473614; doi:10.1371/journal.ppat.1010752)
Supplement: S1 Table — (PDF) [file ppat.1010752.s004.pdf]

S1 Table. The primers used in amplifying the Region E and helix A and/or helix B of BMV, as well as various helix B motifs of CMV 1a, CCMV 1a, HEV ORF1, and RuV P150.

Name	Sequence (NAs in lower case are viral sequences)	Note
VTXW1984	ACTTTAACTGCAGTTTAATTAATGtcgtcgactgttattattaacgg	sense primer for BMV 1a Region E
VTXW1985	CCACCTACATCGCGGCCGCAcggtacagaatcttgctcaaa	antisense primer for BMV 1a Region E
VTXW1986	ACTTTAACTGCAGTTTAATTAATGgtggcctttgctttgactttg	Sense primer for BMV 1a helix A
VTXW1987	CCACCTACATCGCGGCCGCAtgaatctccaccccaccaaaa	antisense primer for BMV 1a helix B
VTXW1990	CCACCTACATCGCGGCCGCAggccgtaagcttttcgtact	antisense primer for BMV 1a helix A
VTXW1992	ACTTTAACTGCAGTTTAATTAATGtggaaaggttggtgccatca	sense primer for BMV 1a helix B
VTXW2475	CTATACTTTAACTGCAGTTTAATTAatggaggttgagcatgcccagaaa	sense primer for HEV ORF1 helix B
VTXW2476	ACCACCACCACCTACATCGCGGCCGCAagacttctcaaatagccaact	antisense primer for HEV ORF1 helix B
VTXW2567	CTATACTTTAACTGCAGTTTAATTAATGtggaagggctgggtcaaccac	sense primer for CCMV 1a helix B
VTXW2568	ACCACCACCACCTACATCGCGGCCGCAcgtacttcctccccaccaaaa	antisense primer for CCMV 1a helix B
VTXW2569	CTATACTTTAACTGCAGTTTAATTAatgatgtatgatatgtggaatgcttcgagtatt	sense primer for CMV 1a helix B
VTXW2570	ACCACCACCACCTACATCGCGGCCGCAaacaacactggagaaaaa	antisense primer for CMV 1a helix B
VTXW3036	atggacaccatttgggacgccattaagcggttcctcggtTGCGGCCGCGATGTAGGTGGTGGTG	sense primer for RuV P150 helix B
VTXW3037	accgaggaaccgcttaatggcgtcccaaatggtgtccatTAATTAAACTGCAGTTAAAGTATAGA	antisense primer for RuV P150 helix B
